# Ongoing Movement of the Hermit Warbler X Townsend's Warbler Hybrid Zone

**DOI:** 10.1371/journal.pone.0014164

**Published:** 2010-11-30

**Authors:** Meade Krosby, Sievert Rohwer

**Affiliations:** Department of Biology and Burke Museum, University of Washington, Seattle, Washington, United States of America; University of Western Ontario, Canada

## Abstract

**Background:**

Movements of hybrid zones – areas of overlap and interbreeding between species – are difficult to document empirically. This is true because moving hybrid zones are expected to be rare, and because movement may proceed too slowly to be measured directly. Townsend's warblers (*Dendroica townsendi*) hybridize with hermit warblers (*D. occidentalis*) where their ranges overlap in Washington and Oregon. Previous morphological, behavioral, and genetic studies of this hybrid zone suggest that it has been steadily moving into the geographical range of hermit warblers, with the more aggressive Townsend's warblers replacing hermit warblers along ∼2000 km of the Pacific coast of Canada and Alaska. Ongoing movement of the zone, however, has yet to be empirically demonstrated.

**Methodology/Principal Findings:**

We compared recently sampled hybrid zone specimens to those collected 10–20 years earlier, to test directly the long-standing hypothesis of hybrid zone movement between these species. Newly sampled specimens were more Townsend's-like than historical specimens, consistent with ongoing movement of the zone into the geographical range of hermit warblers.

**Conclusions/Significance:**

While movement of a hybrid zone may be explained by several possible mechanisms, in this case a wealth of existing evidence suggests that movement is being driven by the competitive displacement of hermit warblers by Townsend's warblers. That no ecological differences have been found between these species, and that replacement of hermit warblers by Townsend's warblers is proceeding downward in latitude and elevation – opposite the directions of range shifts predicted by recent climate change – further support that this movement is not being driven by alternative environmental factors. If the mechanism of competitive displacement is correct, whether this process will ultimately lead to the extinction of hermit warblers will depend on the continued maintenance of the dramatic competitive asymmetry observed between the species.

## Introduction

Empirical documentation of moving hybrid zones – areas of overlap and interbreeding between species – is relatively rare [1]. This is likely because hybrid zones are most often stable over time [2], and because movement, when it does occur, may proceed too slowly to be measured directly [2]. On the other hand, hybrid zone movement due to dramatic competitive asymmetries may lead to rapid extinction of the competitively inferior species [1], [3], so that direct observation is precluded by the ephemeral nature of such events. Recent studies of moving hybrid zones have demonstrated their utility for studying such competitive replacements in action: competitive interactions can be measured directly at hybrid zones, while the extent of historical replacement can sometimes be revealed by genetic signatures of hybrid zone movement.

The best example of this may be provided by hermit warblers and Townsend's warblers, sister species that interbreed in a series of narrow hybrid zones in Washington and Oregon [4], [5]. Previous research suggests that their hybrid zone has shifted ∼2000 km southward along the Pacific coast following secondary contact far to the north, leaving a genetic wake of hermit mitochondrial DNA behind in coastal populations of Townsend's warblers as a footprint of the original extent of the hermit range [4], [5]. No ecological differences between these two species have been found where they coexist in and near the hybrid zone [6]. At the same time, experimental field studies have revealed significant differences in competitive ability: Townsend's males have higher testosterone levels [7] and are much more aggressive than hermit males [8]; they are also more successful at attracting mates and maintaining territories [9]. This body of information suggests that the massive geographic replacement of hermit warblers by Townsend's that is likely to have occurred along the northern Pacific coast has been driven by the competitive superiority of male Townsend's warblers.

To test directly whether the hybrid zone movement between hermit warbler and Townsend's warbler inferred from these earlier studies continues today, we compared the morphological character scores of recently collected hybrid zone specimens to historical specimens collected at the same sites. If the hybrid zone has moved into the hermit geographical range (i.e., if Townsend's warblers are continuing to replace hermit warblers where they overlap), then the new samples should be more Townsend's-like.

## Materials and Methods

### Ethics Statement

This study was approved by the University of Washington Institutional Animal Care and Use Committee (IACUC number 2618-09).

### Methods

**Figure 1:**
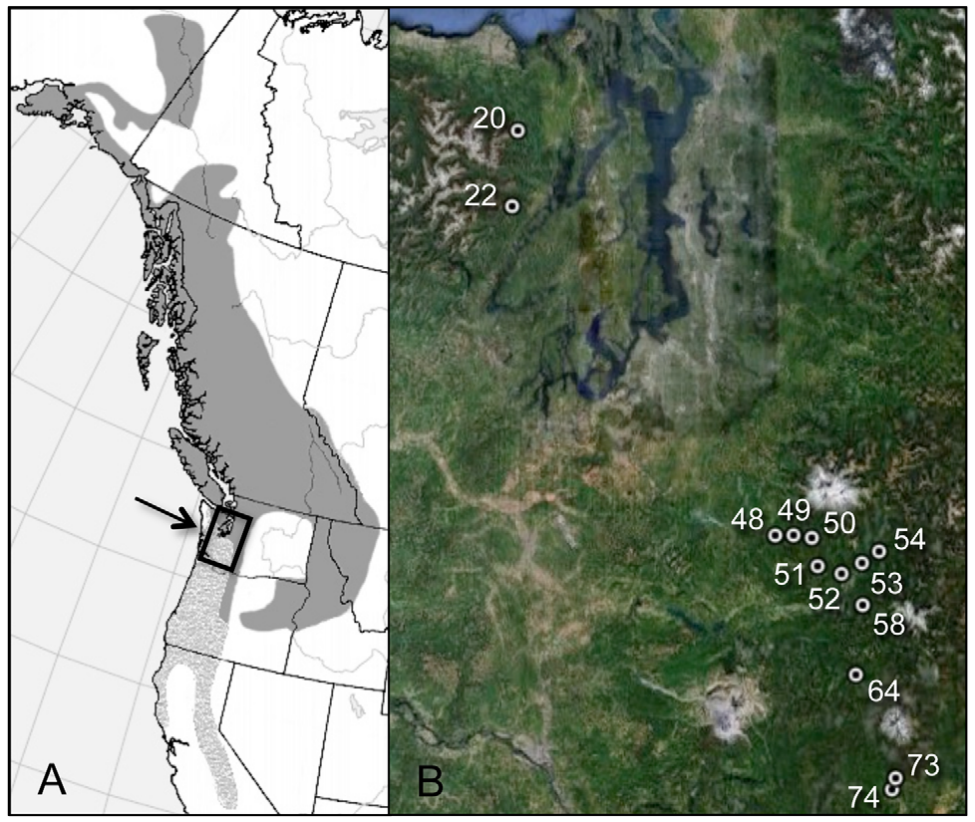
a) Geographical ranges of hermit warbler (light gray) and Townsend's warbler (dark gray) in western North America, with sampling area shown in inset. b) Re-sampling localities used to measure changes in morphological scores between historical and new samples.

In 2005–2008 we re-sampled 13 hybrid zones sites in the Cascade and Olympic Mountains of Washington that had been sampled in 1987–1994 by Rohwer and Wood [10] (Figure 1, Table S1). Zone movement over such a short period of time should be barely detectable, so we focused re-sampling on localities near the phenotypic centers of the hybrid zones, where individuals appear to be, on average, half hermit and half Townsend's, and the rate of change should be greatest.

We limited re-sampling to the two Washington hybrid zones because they had been originally sampled up to ten years earlier than the Oregon zone and included far more sites near the phenotypic centers of zones [10], [11]. We re-sampled all historically sampled sites near the phenotypic centers of these two zones, totaling 11 sites in the Washington Cascade Mountains hybrid zone, and 2 sites in the Olympic Mountains hybrid zone. Historical specimens from several sites were temporally and/or spatially sub-sampled (prior to phenotypic scoring) to maximize time elapsed between historical and new specimens and to ensure that re-sampling took place at the same exact location. For example, for sites including a broad temporal range of historical specimens, only older specimens were included in our study. Also, several sites included historical specimens collected at localities that had since become inaccessible due to residential development, logging, or road closures; such historical specimens were excluded, and re-sampling was limited to historical localities where undisturbed habitat remained undisturbed. Both historical and new specimens were collected using recordings of hermit and Townsend's songs to attract birds, following the methods of Rohwer and Wood [10]. To exclude migrants (i.e., to limit sampling to locally breeding birds), neither historical nor new specimens were collected before May 25^th^. Because collecting methods were the same for both historical and new samples, any potential biases in collecting methods should not influence our results.

Both historical (N = 154) and new (N = 127) specimens were scored for 7 plumage characters to generate a hybrid index ranging from 0 (hermit warbler extreme) to 1 (Townsend's warbler extreme) following Rohwer & Wood [10]. Historical specimens were rescored (by M.K.) to eliminate potential observer differences in assigning plumage scores. Mean hybrid index was calculated for historical and new samples from each site, and an increase in means across all sites was tested using a sign test (one-tailed). The sign test is non-parametric and conservative, appropriate for the statistical challenge of detecting the small phenotypic change expected over such a short period of time, assuming our samples are independent. To confirm the sign test's assumption of independence among samples, we used Moran's I to test for spatial autocorrelation among sites.

## Results

**Figure 2:**
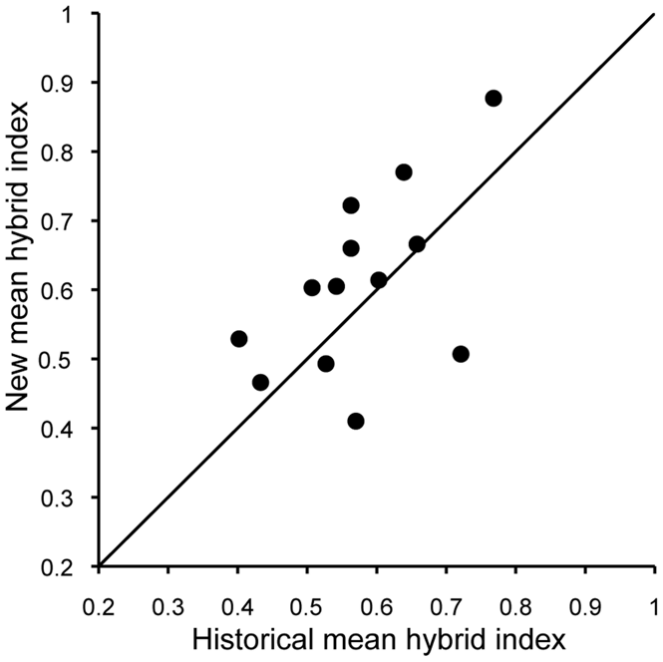
Mean hybrid index of new specimens versus mean hybrid index of historical specimens at 13 sites in the hybrid zones between hermit and Townsend's warblers. Line through origin represents no change over time; points above the diagonal reflect sites that have become more Townsend's-like, and points below the diagonal reflect sites that have become more hermit-like.

Average hybrid indices at re-sampled hybrid zone sites have become significantly more Townsend's-like since the earlier specimens were collected (Figure 2; *P* = 0.046). Change in average hybrid index among sites was not found to be spatially autocorrelated (Moran's I = −0.13, *P* = 0.82), confirming independence among samples.

## Discussion

There are several possible mechanisms that could explain the hybrid zone movement we document here. First, it is possible that this movement has resulted from a stochastic shift of the hermit x Townsend's hybrid zone. However, there is no theoretical foundation for such random movements of hybrid zones, and it seems unlikely that stochastic processes would lead a hybrid zone to move in a single direction over thousands of kilometers, as previous genetic studies have suggested for this zone [4], [5]. Rather, hybrid zone movement is expected to be driven most often by demographic or fitness differences between species, and is therefore expected to be transient, ultimately halting at demographic troughs or when one of the species becomes extinct [2], [3]. Second, it is possible that changes in environmental factors, such as climate, might cause the ranges of both species to move in the same direction, thus also causing the hybrid zone to move [e.g., 12]. However, the hybrid zones between hermit and Townsend's warblers are moving downward in latitude and elevation, opposite to the directions predicted by observed climate change driven range shifts in other taxa [13], [14]. Furthermore, experimental studies have demonstrated a lack of ecological differences between the species [6], making other environmental mechanisms unlikely. Finally, it is possible that the observed movement is being driven by fitness differences between the species. We favor this explanation because it is consistent with previous ecological and behavioral studies demonstrating the competitive superiority of Townsend's warblers [7], [8], [9]. These studies, together with genetic and morphological evidence for both historical and current zone movement toward the hermit geographical range [4], [5], [10], led us to predict *a priori* not only that the zone should be moving, but that it should be moving in the direction observed. Competitive displacement of hermit warblers by Townsend's warblers therefore offers the most likely explanation for the observed movement of their hybrid zone.

How could Townsend's warblers so successfully continue to displace hermit warblers by expanding their range in directions opposite to those predicted by climate change? The wake of hermit warbler mitochondria found in populations of coastal Townsend's far north of their present-day hybrid zones suggests that Townsend's warblers from drier forests east of the coastal ranges have historically been able to replace hermit warblers from moist coastal forests [4], [5]. We thus see little reason to expect that the encroachment of Townsend's warblers might be stalled by any habitat differences encountered in the remaining hermit breeding range.

It is, however, surprising that Townsend's warblers should be so much more aggressive than hermit warblers, their closely related sister species. Rohwer et al. [4] suggested that the hyper-aggressiveness of Townsend's warblers evolved as a response to their summer range being small relative to their winter range at the last glacial maximum. Such an asymmetry in summer and winter range size would lead to intense competition for breeding territories. Further, this range size asymmetry was larger for Townsend's warblers than for hermit warblers [4], which would lead to the observed difference in aggressiveness between the species. Recent genetic work is consistent with this posited difference in their historical population sizes [5]. But as Townsend's warblers have displaced hermit warblers across their breeding range, the historical asymmetries in the relative sizes of their respective winter and summer ranges have been reversed. This, in turn, could eventually lead to the evolution of greater territorial aggression in hermit warblers and less territorial aggression in Townsend's warblers, and ultimately halt the hybrid zone movement we document here.

## Supporting Information

Table S1 Hybrid zone re-sampling: summary of localities and specimens collected.
(0.05 MB DOC)
