# Supplementary material for: Ongoing Movement of the Hermit Warbler X Townsend's Warbler Hybrid Zone
Source: PLoS One. 2010 Nov 30;5(11):e14164. doi: 10.1371/journal.pone.0014164 (PMC2994780; doi:10.1371/journal.pone.0014164)
Supplement: Table S1 — Hybrid zone re-sampling: summary of localities and specimens collected. (0.05 MB DOC) [file pone.0014164.s001.doc]

Table S1. Hybrid zone re-sampling: summary of localities and specimens collected.

| Site | Latitude/ longitude | Sampling date | Sample size | Mean hybrid index (range) | Specimen museum numbers (UWBM^1^) |
| --- | --- | --- | --- | --- | --- |
| 20 | 47.8578° N 123.0260° W | 1986-1987 | 6 | 0.542 (0.385-0.768) | 50839-40, 51356-58, 51360 |
|  |  | 2008 | 6 | 0.605 (0.403-0.872) | 87559-64 |
| 22 | 47.6500° N 123.0485° W | 1987 | 7 | 0.402 (0.175-0.645) | 51349-55 |
|  |  | 2008 | 8 | 0.529 (0.388-0.741) | 87539-46 |
| 48 | 46.7367° N 121.9775° W | 1986-1987 | 7 | 0.570 (0.246-0.866) | 50845-46, 50859-60, 52343-45 |
|  |  | 2007-2008 | 7 | 0.410 (0.150-0.706) | 85173, 87180-84, 87557 |
| 49 | 46.7368° N 121.9040° W | 1986-1987 | 8 | 0.527 (0.276-0.914) | 50847-48, 52336-41 |
|  |  | 2008 | 8 | 0.493 (0.246-0.936) | 87172-79 |
| 50 | 46.7290° N 121.8302° W | 1986-1989 | 8 | 0.563 (0.103-0.916) | 50849, 52290-96, 154786* |
|  |  | 2007 | 8 | 0.660 (0.322-0.843) | 87185-88, 87190-92 |
| 51 | 46.6503° N 121.8080° W | 1987-1989 | 17 | 0.563 (0.139-0.907) | 52296-97, 52299, 52300-07, 52348-50, 52352, 154787*, 154789* |
|  |  | 2007 | 10 | 0.722 (0.427-0.951) | 87193-202 |
| 52 | 46.6287° N 121.7120° W | 1987 | 15 | 0.658 (0.268-0.930) | 52308, 52321-22, 52324-35 |
|  |  | 2006 | 12 | 0.666 (0.119-0.948) | 84790-96, 84839-40, 84844, 84846, 84852 |
| 53 | 46.6575° N 121.6290° W | 1987 | 17 | 0.639 (0.216-0.917) | 52409-425 |
|  |  | 2006 | 12 | 0.770 (0.443-0.940) | 84797-800, 84802-03, 84848-51, 84853 |
| 54 | 46.6890° N 121.5592° W | 1986-1987 | 15 | 0.768 (0.569-0.926) | 50851, 50895-900, 52359, 52365-66, 52405-08, 52376 |
|  |  | 2008 | 11 | 0.877 (0.721-0.984) | 87515, 87817-26 |
| 58 | 46.5413° N 121.6280° W | 1987 | 9 | 0.721 (0.403-0.960) | 52309-17 |
|  |  | 2008 | 10 | 0.507 (0.194-0.830) | 87547-56 |
| 64 | 46.3485° N 121.6583° W | 1987 | 14 | 0.433 (0.119-0.884) | 52453-56, 52482-87, 52489-92 |
|  |  | 2008 | 12 | 0.466 (0.162-0.908) | 87527-38 |
| 73 | 46.0578° N 121.5008° W | 1994 | 11 | 0.603 (0.192-0.956) | 49149-59 |
|  |  | 2005 | 14 | 0.614 (0.295-0.911) | 80771, 80787, 80789, 80792-93, 80802-10 |
| 74 | 46.0258° N 121.5158° W | 1994 | 20 | 0.507 (0.094-0.926) | 49918-34, 49936, 49939-40, 49942 |
|  |  | 2005 | 9 | 0.603 (0.280-0.876) | 80770, 80778-79, 80781, 80795, 80812, 80818-20 |

^1^University of Washington Burke Museum; *specimens from the Louisiana State University Museum of Natural Science.
